# Notes Written by the Patient Whose Case Was Reported by Dr. Ruschenberger, in Our Last Number

**Published:** 1842-01-15

**Authors:** 


// MEDICAL EXAMINER.

NEW SERIES.

No. 3.] PHILADELPHIA, JAN. 15, 1842. [Vol. I.

### ORIGINAL COMMUNICATIONS.

### *Notes written by the patient whose case was reported by* Dr. Ruschenberger, *in our last number.*

The following short notes of this case, were made by the patient himself, and are given in his own words.

*Oct.* 18th, the day of the operation. The *cutting* less painful, and sawing more so than I had anticipated, especially on some portions of the bone, which were more sensitive than other portions. The use of the hammer and chisel to separate extraneous masses of bone, excessively shocking to the nervous system. It occupied rather more than one hour ere I was placed in bed and the limb adjusted, and three-quarters of an hour under the actual operation.

On being put to bed, and the wound dressed, I had little or no pain, and in every sense became comfortable. What pain there was occurred at intervals of half to three-fourths of an hour, lasting only a few minutes, of a burning sensation rather than what I should call acute pain, and which seemed to linger about the exterior. Pulse rather full till nine P. M., when a healthy reaction took place. No symptoms of fever. The night was sleepless; little pain, but continued thirst.

*2d day.* The pains diminishing, and the intervals between them increased. Good spirits: no fever. An acute soreness in the abdomen, with excessive pain in the back, doubtless the effect of position. Had an hour's sleep. During the night obtained three hours sleep; but suffered greatly with pain in the abdomen and from thirst.

*3d day.* Little or no pain in the limb. Soreness in back and abdomen continuing; spirits good; no fever; little appetite. During the night had three hours sleep; much thirst; and always more nervous during the night than day.

*4th day.* Pain in abdomen and back very much abated, and the whole system more agreeable than heretofore. Appetite better. No fever. To-day the wound was exposed, and pronounced to have a healthy appearance. Though the day was agreeable, yet the night was the most irksome and painful of any previous to it, caused by a spasmodic attack in the limb at eleven P. M., and repeated several times during the night, occasioning an unusual flow of blood, creating pricking sensations about the wound and in the calf, from which I was relieved when the wound was dressed in the morning. It was remarked to-day that the muscles seemed to have accommodated themselves easily to their present and more natural position, from which they had been diverted just *two years and ten months;* and the bones retained in the desired position by very slight means.

*5th day.* After the dressing the limb became easy: the *back* and *abdomen* are entirely relieved from pain at last. No fever, and spirits good. Several spasms occurred.

Had five hours sleep and no spasm.

*6th day.* No actual pain, but the wound is becoming extremely sensitive to touch or motion. The foot benumbed and heavy, and subject to frequent change from heat to cold, and vice versa. Digestion good. Four hours sleep and no spasm.

*7th day.* The sensitiveness of the wound increasing, but still appears healthy; (what the doctors call beautiful.) The foot becoming more and more weighty, but has not swollen in the least.

Had four hours sleep at night; spasms abated.

*8th day.* Differs in no degree from the preceding one

*9th day.* The wound has now become unbearably sensitive to the slightest touch or motion—ridiculously so. The spring of the floor, occasioned by a person walking across the room, and even the jar communicated to the building by carriages driving along the paved streets, affecting (if not actually afflicting) the wounded parts. In the first instance the pain was confined to the exterior wound, but now the interior has more than its share. Especially where the extraneous bones or bridges were removed is the seat of inconvenience.

*10th day.* No change in the last twenty-four hours.

*11th day.* The same as the preceding twenty-four hours.

*12th day.* No alteration in the sensitiveness of the wounded parts. At 5 P. M. was seized with a severe pain in the foot, as if all the bones in it had been crushed by some weighty mass. At six it became excruciating; caused it to be rubbed with oil of camphor, which allayed the pain almost immediately.

*13th day.* Extreme sensitiveness of the wound *somewhat* abated; otherwise no pain.

*14th day.* The transitions from heat to cold, &c., benumbed feeling, and sense of weight in the foot, mentioned on the sixth day, have continued up to this time, but at length are abating, together with the sensitiveness in the region of the wound.

From the second to the end of the third week, no new symptoms. No swelling. Good health. All inconveniences gradually diminishing. The union of the bones considered to have occurred unusually rapid during this week. On the twenty-first day it was considered tolerably firm. The only pain and inconvenience to which I am now subjected, are from the application of *splints*, *compresses*, and such means as are usual for retaining the bones in their proper place.

To the end of the fourth week little or no pain, or other inconvenience consequent upon the operation. The union of the bones has become so firm as to admit of the limb being rolled about the pillow, and the wound healed, except in one point, where it is frequently probed, a fragment of bone being about to detach itself.

To the end of the fifth week, the union hardening perceptibly each day. Left the bed several times this week, for a few minutes, to receive a cold shower upon the limb. On the fortieth day arose from the bed on crutches, and in four days thereafter the splints and bandages were dispensed with.

REMARKS.

During my confinement to bed, the pulse ranged from 70 to 87; and sometimes, from exciting causes, as high, temporarily, as 93, But my pulse is usually rapid, and easily excited. My appetite did not vary from its customary moderate standard.

Entertaining no anxiety for the result, my mind was calm; and I was even satisfied with my confinement and almost torpid state of body.

It was remarked that the greatest degree of uneasiness, pains, spasms, and other annoyances incident to my condition, always occurred just as I was about to compose myself to sleep.

In the foregoing account, where I have given three, four, or five hours sleep, I would explain that I seldom had an unbroken repose for that time; that only on one occasion I had an hour's sleep in daylight; and that the night of the day on which I quitted the bed was the first I enjoyed for the entire night. In health, however, I am accustomed to little sleep—seldom more than five hours.

